# Vaccine Hesitancy Toward Dengue Immunization Among Indonesian Office Workers: A Cross-Sectional Study of Perceptions, Barriers, and Trust Factors

**DOI:** 10.3390/vaccines13121178

**Published:** 2025-11-21

**Authors:** Theresia Santi, Ridwansyah Ridwansyah, Veli Sungono, Natalia Widjaya, Keinata Nabila Euqenekim, Cessya Prianyanta, Sri Rezeki S. Hadinegoro, Budi Setiabudiawan, Juandy Jo

**Affiliations:** 1 Faculty of Medicine, President University, Bekasi Regency 17530, Indonesia; theresia.santi@president.ac.id (T.S.); ridwansyah@president.ac.id (R.R.); natalia.widjaja@president.ac.id (N.W.); keinata.euqenekim@student.president.ac.id (K.N.E.); cessya.prianyanta@student.president.ac.id (C.P.); budi.setiabudiawan@president.ac.id (B.S.); 2 Faculty of Medicine, Universitas Pelita Harapan, Tangerang 15811, Indonesia; veli.sungono@uph.edu; 3 Department of Child Health, Faculty of Medicine, Universitas Indonesia, Cipto Mangunkusumo Hospital, Jakarta 10430, Indonesia; shadinegoro46@gmail.com; 4 Department of Child Health, Faculty of Medicine, Universitas Padjajaran, Hasan Sadikin General Hospital, Bandung 40161, Indonesia; 5 Department of Biology, Faculty of Health Sciences, Universitas Pelita Harapan, Tangerang 15811, Indonesia; 6 Mochtar Riady Institute for Nanotechnology, Universitas Pelita Harapan, Tangerang 15811, Indonesia

**Keywords:** dengue, vaccine, hesitancy, willingness-to-pay, office workers

## Abstract

Background/Objectives: In the absence of specific antiviral therapy for dengue viral infection, vaccination remains the most effective preventive measure. Two dengue vaccines have been licensed in Indonesia; however, concerns regarding vaccine hesitancy persist. This study aimed to assess dengue vaccine hesitancy among Indonesian office workers, comprising healthcare and non-healthcare workers. Methods: A cross-sectional study with an online survey was conducted between February 1 and April 30, 2025. Eligible participants were adults (≥18 years) employed in office-based settings, including healthcare facilities. Questionnaires were disseminated through company management teams and included 37 items on demographic characteristics, vaccination intentions, and underlying motivations. Data were analyzed to identify determinants of vaccine hesitancy. Results: A total of 377 respondents participated, the majority of whom were from West Java (335; 88.9%). One-third of respondents reported uncertainty regarding dengue vaccination (33.4% “not sure”), which was paralleled by hesitancy to pay for vaccination (43.2% “not sure”). Multivariable logistic regression analysis identified five significant determinants of vaccine hesitancy, with willingness-to-pay emerging as the strongest factor (β coefficient = 2.024; OR = 7.57; 95% CI = 4.06–14.10; *p*-value < 0.01). Conclusions: Approximately one-third of the surveyed Indonesian office workers exhibited hesitancy toward dengue vaccination. Willingness-to-pay was the most influential determinant of vaccine acceptance. Targeted strategies to address financial concerns and improve confidence in dengue vaccination are essential for strengthening workforce protection and national preparedness against dengue outbreaks.

## 1. Introduction

Dengue is among the most significant yet under-recognized tropical diseases globally, with its incidence increasing more than 30-fold in recent decades [1,2]. This dramatic rise is largely attributed to the widespread expansion of its primary vectors, *Aedes aegypti* and, to a lesser extent, *Aedes albopictus*, alongside the dissemination of the four dengue virus serotypes (DENV-1 to DENV-4). Dengue transmission now occurs across vast geographical regions, including the Eastern Mediterranean, Americas, Southeast Asia, Western Pacific, and Africa, with emerging outbreaks reported in previously non-endemic areas, such as parts of the United States and Europe [3,4]. Infection by any DENV serotype can result in a wide range of clinical manifestations, of which both the timing and sequence of infections significantly influence disease severity [1,2]. Based on the 2009 classification of the World Health Organization (WHO), dengue is classified as either dengue (with or without warning signs) or severe dengue [5]. Between 1990 and 2021, global dengue cases rose from 26.45 million to 58.96 million, while dengue-related deaths more than doubled from 14,315 to 29,075. Over the same period, disability-adjusted life years due to dengue increased from 1.25 million to 2.08 million, with the greatest burden observed in South Asia, Southeast Asia, and tropical Latin America [3]. It is predicted that by 2030, the incidence and mortality rates of dengue in adults will increase to 872.94 per 100,000 population and 0.24 per 100,000 population, respectively [4].

Indonesia, one of the largest dengue-endemic countries in Southeast Asia, has experienced a substantial increment of dengue cases. National surveillance data between 1968 and 2017 had indicated a substantial increase in incidence of severe dengue (from 0.05 cases per 100,000 person-years in 1968 to 77.96 in 2016) accompanied by cyclical outbreaks occurring approximately every 6–8 years. Major epidemics of dengue in Indonesia had occurred in 1973, 1988, 1998, 2009, and 2016. In 2017, a total of 59,047 DHF cases and 444 deaths had been reported, corresponding to an incidence rate of 22.55 per 100,000 population and a case-fatality rate (CFR) of 0.75%. By the 17th epidemiological week in 2024, a total of 88,593 dengue cases and 621 deaths had been reported, a threefold increase compared to the same period in 2023 [6]. While non-endemic regions (e.g., Papua and West Papua) had experienced sporadic outbreaks with relatively higher fatality rates, West Java had continuously reported the highest average number of severe dengue cases annually [7]. Spatial analysis had revealed concentrated dengue hotspots in urban centers across West Java, where high population density may amplify transmission risk [8]. A distinct seasonal pattern is evident, with dengue cases peaking during the rainy season (November–March) and declining during the dry season (April–October) [8,9]. This underscores the significant influence of both climatic variability and demographic factors on dynamics of dengue transmission in Indonesia.

Annual economic loss in Indonesia due to dengue had reached approximately USD 323 million due to dengue between 2001 and 2010 [10]. A recent study in 2015 provided a more comprehensive assessment of dengue-related costs across three Indonesian provinces, of which the estimated cost per dengue case was USD 791 in Yogyakarta, USD 1241 in Bali, and USD 1250 in Jakarta [11]. Using these provincial estimates and an empirically derived epidemiological expansion factor, the national economic burden of dengue in 2015 was projected at USD 381 million, comprising USD 355 million for hospitalized cases and USD 26 million for outpatient care [11]. The risk of significant financial burden of dengue on both the Indonesian healthcare system and society highlight an urgent need for evidence-based health policy and targeted prevention strategies.

In the absence of specific antiviral therapy against the dengue virus, current disease control strategies rely primarily on supportive clinical care and vector control with limited and inconsistent success in curbing dengue transmission [12]. Vaccination indeed emerges as a crucial complementary strategy to reduce the disease burden [13,14]. The first licensed dengue vaccine, CYD-TDV (a live-attenuated tetravalent vaccine based on a yellow fever 17D backbone) was introduced in 2015. However, it was later restricted to individuals with prior dengue exposure due to an elevated risk of severe disease in seronegative recipients [15,16]. A second vaccine, TAK-003 (a live-attenuated tetravalent vaccine based on a DENV-2 backbone), was approved by the WHO for use in children aged 6 to 16 years living in endemic areas [17]. Its regulatory approvals currently vary by region: while the European Union permits its use in children as young as four, Brazil and Indonesia have approved it for individuals aged 4 to 60 years and 6 to 45 years, respectively [18,19,20]. Although TAK-003 has not shown any evidence of antibody-dependent enhancement up to 4.5 years post-vaccination, its efficacy was highest against DENV-2 but was lower for DENV-3 and DENV-4 among seronegative individuals [21].

However, vaccine hesitancy indeed remains a major barrier to achieving high immunization coverage, particularly in dengue-endemic countries [22]. To address this challenge, we conducted a cross-sectional study using an online survey to investigate determinants of dengue vaccine acceptance among Indonesian office workers, a population that is eligible for the recently approved TAK-003 vaccine for Indonesians. The health of this workforce is closely tied to national socioeconomic stability, as dengue infections can lead to absenteeism, reduced productivity, and substantial economic losses for both individuals and employers [11,23,24]. As primary wage earners, office workers’ health-related decisions can also substantially affect the health of their family members [25,26]. Therefore, Indonesian office workers were studied to identify determinants of dengue vaccine uptake among adults whose health is vital to the societal and economic well-being of the overall population.

## 2. Materials and Methods

### 2.1. Study Design and Subject

A cross-sectional study with an online survey was conducted between 1 February and 30 April 2025. Companies were invited to participate in this survey without using a specific sampling frame or strategy. Participant recruitment was conducted through the annual National Occupational Safety and Health Month event organized at the President University, which was strategically situated within a major industrial area in West Java. The event was attended by senior management and human resources department representatives from approximately 1500 companies operating within the industrial zone. Additionally, companies with branches located outside of West Java were approached to obtain study participants from other provinces of Indonesia. Companies varied in size, employing between 50 and 1500 workers. With permission from company representatives, all employees were invited to participate in the study. The convenience sampling method was employed to recruit participants, in which online questionnaires were distributed to companies’ management teams, who subsequently disseminated the forms to their employees. The online survey link was distributed to company leaders and HRD representatives, who subsequently shared it with their employees via WhatsApp. To improve participation, reminders were sent to the companies at three intervals: on the initial day of distribution, seven days later, and one month after the initial invitation. A final reminder was sent seven days before the survey closed on 30 April 2025. The sample size was calculated using the Slovin’s formula, based on the number of dependent variables to be explored, yielding a minimum of 100 subjects. The recruited participants were located across Indonesia, including West Java, Jakarta, South Sumatra, East Kalimantan, Central Sulawesi, and Papua. The inclusion criteria were participants aged over 18 years old and currently employed in office-based settings, including healthcare facilities. The exclusion criterion was individuals who were unwilling to provide informed consent. Prior to answering the questionnaire, all participants were given written explanations and informed consents were obtained. Only one response per WhatsApp number was recorded to exclude data duplicity.

To improve quality of reporting, the STROBE (Strengthening the Reporting of Observational Studies in Epidemiology) guidelines were followed [27]. Data collection employed structured questionnaires designed to assess various demographic and attitudinal factors influencing dengue vaccine uptake among occupational groups in endemic regions. The survey instrument was evaluated for validity and reliability prior to data collection. Content validity was established by two experts, i.e., the Chair of the Indonesian National Immunization Technical Advisory Group (S.R.S.H.; provided feedback on questionnaire relevance and structure), and an occupational health physician at the President University (assessed the clarity and applicability of the questions). Reliability was examined through a pilot test involving 30 participants, with internal consistency measured using Cronbach’s alpha [28]. The analysis yielded a Cronbach’s alpha value of 0.773, indicating acceptable reliability of the instrument. The questionnaire, which took approximately 10 min to complete, comprised 37 items addressing demographic variables, vaccination intentions regarding dengue, and underlying motivations (Supplementary File). The questionnaire was presented in Bahasa Indonesia, consisting of four parts. The first part comprised the demographic questions (i.e., name, date of birth, age, gender, education level, occupation, and income level). The second part consisted of additional questions for variables associated with intention to vaccinate, such as personal experience of contracting dengue, familial history of dengue, concerns pertaining to dengue, and consideration of the vaccine’s cost. The third part was inspired by the study by Chaudary et al. [29] and adapted for Indonesia’s context, in which this section evaluated participant knowledge about dengue and its vaccines. Each correct answer was assigned a score of 1, and each incorrect answer a score of 0. The total score for this part was calculated, with scores below 6 classified as poor and scores of 6 or above classified as good. The fourth part on attitudes to prevent dengue infection was assessed using a five-point Likert scale, ranging from ‘strongly agree,’ ‘agree,’ ‘not sure’, ‘disagree,’ to ‘strongly disagree’ [30]. A response of ‘strongly agree’ was assigned a score of 5, and ‘strongly disagree’ a score of 1, with intermediate responses scored as follows: ‘agree’ = 4, ‘not sure’ = 3, and ‘disagree’ = 2. The total score for this part was calculated, with scores below 35 classified as poor and scores of 35 or above classified as good.

### 2.2. Statistical Analyses

Descriptive statistics were used to summarize respondent characteristics. Categorical variables (i.e., sex, education level, income, occupation, personal experience of dengue, family history of dengue, knowledge level, attitude, intention to vaccinate, and opinion on paying the vaccine) were presented as frequencies and percentages. Continuous variables (e.g., age) were described using means or medians, depending on the normality of their distribution. Univariate analysis, including frequency distributions, was performed to describe the sample profile. Bivariate associations between variables and intention to receive the dengue vaccine were assessed using Chi-Square test. Variables with *p*-value < 0.25 in bivariate analysis were entered into a multivariable logistic regression model using a stepwise forward selection approach to determine independent determinants of vaccination willingness. Statistical significance was defined as *p*-value < 0.05. All statistical analyses were conducted using STATA IC version 16.1 (StataCorp LLC, College Station, TX, USA).

### 2.3. Ethical Approval

The study protocol was approved by the Institutional Review Board of Bekasi Regional General Hospital (KP.800.1.10.4/886/RSUD/2025).

## 3. Results

This study recruited 377 Indonesian office workers (Figure S1); the most respondents were in West Java (335; 88.9%), while the least were in Central Sulawesi (2; 0.5%) and in Papua (2; 0.5%). As shown in Table 1, the mean age of the respondents was 38.2 years (range 18–60 years old; standard deviation of 9.7). The proportions of female and male respondents were 57.3% (n = 216) and 42.7% (n = 161), respectively. Most participants had a university degree (87.3%) and the majority were non-healthcare workers (76.9%). Majority of the cohort claimed that they had good knowledge on dengue and good acceptance toward the dengue vaccine. While majority of participants expressed an intention to vaccinate against dengue (13.3% of ‘strongly agree’ and 49.1% of ‘agree’), a substantial proportion of this cohort remained hesitant (33.4% of ‘not sure’). This was subsequently reflected in the cohort’s opinion of uncertainty about paying to be vaccinated (43.2% of ‘not sure’). Notably, the stratification analysis revealed only minor differences between participants residing in West Java and those from other Indonesian provinces (“Non-West Java”; Table S1), suggesting that the overall trends were consistent across both subgroups.

Factors related to the intention to vaccinate against dengue were subsequently analyzed with bivariate analysis (Table 2). Although the difference was not statistically significant, as expected, a higher proportion of healthcare workers than non-healthcare workers expressed an intention to receive the dengue vaccine (71.3% versus 59.7%). Several factors significantly influenced intention to vaccinate against dengue (*p*-value <0.05), i.e., education level, family history of dengue, knowledge level on dengue, attitude toward dengue vaccine, fear of dengue, worry of vaccine adverse event, and willingness-to-pay. Higher knowledge level (odds ratio/OR of 1.84) and fear of dengue (OR of 4.18) increased an intention to be vaccinated, while larger concern of vaccine adverse events (OR of 3.67) decreased the intention. Of note, attitude toward dengue vaccine (OR of 9.57) and willingness-to-pay to be vaccinated (OR of 8.98) exhibited the strongest positive association with the dengue vaccine’s acceptance in this cohort.

Ten risk factors with *p*-values <0.25 as depicted in Table 2 were selected for further analysis. A multivariable analysis was conducted to identify strong determinants of vaccine hesitancy based on their *p*-value (i.e., <0.05) and large beta coefficient value. The multivariable model was refined by sequentially removing non-significant variables until only the most significant and influential determinant remained. The obtained determinant was also assessed for its clinical and theoretical importance. The final model revealed five variables ranked by their strength of association with vaccine hesitancy (Table 3). The most influential determinant of vaccine hesitancy was willingness-to-pay to be vaccinated, with beta coefficient of 2.024 and odds ratio/OR of 7.57. This suggests that the study participants who were unwilling to pay for the vaccine had approximately 7.6 the odds of being hesitant or uncertain about vaccination, as compared to those who were willing to pay. Of note, most respondents in this study opted for a price under US$6 per vaccine recipient (data not shown). This underscores the significant impact of cost-related concerns as a barrier to vaccine uptake. The second-most influential factor associated with willingness to vaccinate was the participant’s attitude toward the dengue vaccine (OR of 4.17). This indicates that the study participants with a low receptive level had approximately 4.2 times the odds of vaccine hesitancy, as compared to those with a more positive attitude. The third significant factor associated with willingness to vaccinate was the study participant’s education level. Individuals with lower education level exhibited significantly higher odds of vaccine hesitancy, with OR of 3.72. This suggests that respondents with lower education had approximately 3.7 times the odds of being hesitant about vaccination. The fourth and fifth variables were fear of dengue and worry of vaccine’s adverse events. Respondents without fear toward dengue had OR of 2.54, as compared to those with such fear. Similarly, individuals with concern of the dengue vaccine’s adverse events had OR of 2.53 for developing hesitancy to be vaccinated, as compared to those without such concern.

## 4. Discussion

We hereby reported findings of our cross-sectional study during 1 February until 30 April 2025, investigating risk factors that influenced dengue vaccine acceptance among Indonesian office workers. Our results can be summarized into three main points. First, most study participants were based in the province of West Java. This could be partly due to the affiliated institute of most co-authors (including the first author), of which more employees working in companies located in West Java were more familiar with it, and hence were more willing to answer the online questionnaire. Despite the uneven distribution of study participants across Indonesia, it was important to note that, as of 12 June 2025, West Java reported the highest number of dengue cases (n = 17,281) and the highest number of dengue-related mortalities (n = 297) in Indonesia, likely due to its dense population and high human mobility [8,31,32,33]. Arguably, this close interaction with dengue would allow the study participants in West Java to provide better-informed opinions on dengue vaccine hesitancy.

Second, we discovered that while a minority portion of the cohort (n = 16; 4.3%) disagreed to be vaccinated, a substantial portion of the cohort were still having hesitancy to be vaccinated (n = 126; 33.4%). This highlights a significant challenge in persuading enough Indonesians to be vaccinated against dengue for the vaccine to be effective in creating a herd immunity [34,35]. Several published studies on vaccine acceptance in Indonesia indicated that educated individuals with good knowledge on dengue and its vaccine were likely to be vaccinated [22,36,37,38]. These findings were reinforced by studies from other countries, emphasizing that targeted educational interventions and strategic use of social media are crucial to improving dengue awareness and vaccination, particularly among vulnerable populations [20,29,39]. Of note, a personal or family history of dengue infection appeared to heighten individuals’ awareness and motivation to engage in dengue prevention practices, such as controlling vector transmission [20,29,36]. Consistent with this, our findings indicate that prior dengue experience is a significant determinant of reduced vaccine hesitancy (OR of 2.24; 95% confidence interval/CI of 1.38–3.62; *p*-value <0.01), corroborating published evidence [40].

Third, we noted that there were five substantial risk factors associated with dengue vaccine hesitancy in the cohort, i.e., (i) willingness-to-pay to be vaccinated; (ii) attitude toward dengue vaccine; (iii) study participant’s education level; (iv) fear of dengue; and (v) concern of vaccine’s adverse events. Of those, willingness-to-pay was identified as the strongest determinant of dengue vaccine uptake in our cohort (beta coefficient: 2.024; OR: 7.57; 95% CI: 4.06–14.10; *p*-value < 0.01). This suggests that there is a psychological threshold of the vaccine’s price to be accepted by the community and that there is a crucial need for governments to subsidize the price of the dengue vaccine or even to include the vaccine in routine national vaccination programs [13,14,41,42,43]. Various prices regarding willingness-to-pay for the dengue vaccine have been proposed across different countries [36,37,44,45,46,47,48]. A recent meta-analysis on 19 studies in the Americas and Asia had reported that the median of willingness-to-pay was USD 46.7 (95% CI: 25.9–67.5) per vaccine recipient [48]. It is important to emphasize that this reported median price was at least 10 times higher than the reported ones from several cohorts in Indonesia, i.e., USD 2.1–4 per vaccine recipient [36,37,45], or from the ones observed in our study cohort (i.e., under USD 6 per vaccine recipient). This suggests that the role of governmental subsidy in Indonesia in reducing the vaccine’s price would be substantial to increase the willingness of Indonesians to be vaccinated against dengue.

However, lowering the vaccine’s price through governmental subsidy is only a partial solution to this complex issue. Of note, an issue related to dengue vaccine acceptance was the 2017 controversy of Sanofi Pasteur’s Dengvaxia usage in the Philippines [16]. As Dengvaxia was subsequently reported to be unsafe for dengue-naïve individuals, this issue substantially reduced public confidence in the safety and efficacy of dengue and other vaccines in the Philippines [16]. Although there is no specific study analyzing the impact of this controversy to the dengue vaccine’s acceptance in Indonesia, it is likely contributing to the vaccine hesitancy in Indonesia. During its 80 years as an independent nation, Indonesia has encountered various vaccine hesitancy cases, e.g., during the measles and rubella vaccination programs in 2018 [49,50]. Several issues had contributed to the vaccine hesitancy or even reluctance in accepting the free vaccine in that program, ranging from natural disasters in certain parts of Indonesia to the emergence of another outbreak (i.e., vaccine-derived polio in Papua New Guinea) to the communication issues with religious groups [49]. In addition, as the Coronavirus disease 2019 (COVID-19) pandemic has receded and its vaccination has been recognized as a critical measure in reducing the related morbidity and mortality, it is of interest to analyze the factors contributing to vaccine hesitancy against the disease. Although several COVID-19 vaccines were provided free-of-charge to both adults and children in Indonesia, our previous finding on the pediatric population had reported that a substantial proportion of parents (~30%) were unwilling to vaccinate their children [51]. Two main contributing factors were the inability of the Indonesia government to secure mRNA-based COVID-19 vaccines (which were perceived globally as the better option to protect against COVID-19) and its partial successfulness in securing only 80 million doses of CoronaVac (inactivated virus-based) and CoviShield (viral vector-based) vaccines in 2021, covering 40 million of the most at-risk citizens that accounted for only 22% of the total population eligible for vaccination [50]. In addition, a preprint report speculated that the hesitancy to the COVID-19 vaccine in Indonesia was associated with a low trust in the government during the period [52]. Collectively, this indicated that multi-pronged approaches will be required to increase vaccine acceptance among Indonesians, particularly against dengue.

To the best of our knowledge, although numerous studies have examined dengue vaccine hesitancy among adult populations globally [48,53], our study was novel in its focus on Indonesian office workers. This population is of particular interest due to an indoor-biting behavior of *Aedes* mosquitoes [1,2], the importance of primary wage earners in determining health statuses of their family members [11,23,24,25,26], and because many within this group are eligible to receive the TAK-003 vaccine [18,19,20]. Notably, our findings indicate that a substantial proportion of these individuals remain hesitant and would require targeted strategies to increase vaccine acceptance. This was aligned with a recent publication of 600 Indonesian adults aged 21–60 years old, as a part of the Asia Pacific and Latin American Knowledge Attitude Practice study, reporting that only 51% of the cohort was willing to receive the dengue vaccine [54]. Given their role as primary income earners, the office workers may influence the health decisions of their households as well. Therefore, addressing vaccine hesitancy in this group is essential and warrants greater attention from the public health policymakers in Indonesia.

This study had several limitations. While the Indonesian workforce in 2024 reached approximately 143 million individuals [55], our cohort was small (n = 377) and predominantly based in West Java. Consequently, the findings may not adequately represent the broader Indonesian working population. Selection bias may have influenced the results, as participation was restricted to office workers, which are an urban, educated, and economically stable subgroup. Therefore, the study may not capture perspectives or behaviors of informal workers, rural residents, or individuals with lower socioeconomic status. As only completed responses were collected, our findings might be biased because variables influencing non-participants could differ from those influencing the study participants. Additionally, we did not have data on missing responses. In terms of demographic composition, our cohort was slightly younger (mean age of 38 versus 41 years) and had a higher proportion of female respondents (57.3% versus 39.4%) compared to the national workforce in 2024 [55,56]. Moreover, our cohort had a much larger proportion of participants holding a university degree (87.3% versus 13.1%) than the national workforce in 2025 [57], likely reflecting our focus on office-based employment that generally requires a higher education level. These differences further limit the generalizability of our findings. Additionally, the online survey was self-administered without supervision, making it uncertain whether all participants fully comprehended the questionnaire. Voluntary participation may have also attracted individuals who were more health-conscious or more interested in vaccination, potentially leading to an overestimation of vaccine willingness and preventive behaviors. Next, response bias cannot be ruled out. Since the survey was distributed through employers, some respondents may have perceived that their answers could be viewed by supervisors or organizations, thereby encouraging socially desirable responses (e.g., overstating willingness to vaccinate or engagement in dengue prevention activities) rather than reporting their true attitudes and practices. Finally, we did not perform additional, stand-alone statistical analyses to specifically check for confounding or effect modification. Hence, potential confounders might remain unrecognized or unadjusted and possible interactions (where effects differ across subgroups) might go undetected. As our study cohort was considered as a relatively homogenous cohort (defined as adult by the WHO), we also did not include age-based stratification as a potential variable in our bivariate and multivariable analyses. This might introduce any residual confounding bias in our findings.

## 5. Conclusions

This study explored the factors influencing dengue vaccine acceptance among Indonesian office workers, a population with both elevated exposure risk and substantial societal impact. Approximately one-third of the study participants exhibited vaccine hesitancy, with willingness-to-pay emerging as the strongest determinant of vaccine acceptance. Strengthening vaccine acceptance in this workforce is critical for advancing public health and enhancing the national resilience to dengue outbreaks in Indonesia.

## Figures and Tables

**Table 1:** Characteristics of the study participants (n = 377).

Variable	Value
*Age in year* [Mean ± SD; (Min–Max)]	38.2 ± 9.7 (18–60)
*Sex* [n (%)]	
Female	216 (57.3)
Male	161 (42.7)
*Education* [n (%)]	
Elementary-high school	48 (12.7)
University	329 (87.3)
*Income* [n (%)]	
<IDR 5,000,000	93 (24.7)
IDR 5,000,000–10,000,000	193 (51.2)
IDR 10,000,000–25,000,000	66 (17.5)
>IDR 25,000,000	25 (6.6)
*Occupation* [n (%)]	
Non-healthcare worker	290 (76.9)
Healthcare worker	87 (23.1)
*Personal history of dengue* [n (%)]	
No or do not know	284 (75.3)
Yes	93 (24.7)
*Family history of dengue* [n (%)]	
No	259 (68.7)
Yes	118 (31.3)
*Knowledge level of dengue* [n (%)]	
Poor	77 (20.4)
Good	300 (79.6)
*Attitude toward dengue vaccine* [n (%)]	
Poor	64 (17.0)
Good	313 (83.0)
*Intention to vaccinate against dengue* [n (%)]	
Strongly agree	50 (13.3)
Agree	185 (49.1)
Not sure	126 (33.4)
Disagree	10 (2.7)
Strongly disagree	6 (1.6)
*Opinion about paying for the vaccine* [n (%)]	
Strongly agree	19 (4.2)
Agree	132 (35.0)
Not sure	163 (43.2)
Disagree	47 (12.5)
Strongly disagree	16 (4.2)

IDR, Indonesian Rupiah; SD, standard deviation. *Personal history of dengue* refers to respondent’s direct encounter with dengue infection. *Family history of dengue* refers to occurrence of dengue infection among a respondent’s family members. *Knowledge level of dengue* refers to extent and accuracy of a respondent’s understanding on dengue infection. *Attitude toward dengue vaccine* refers to a respondent’s opinion toward receiving the dengue vaccine. *Intention to vaccinate against dengue* refers to a respondent’s willingness to receive the dengue vaccine. *Opinion about paying for the vaccine* refers to a respondent’s attitude, regarding the cost and financial responsibility associated with receiving the dengue vaccine.

**Table 2:** Bivariate analysis on factors associated with intention to vaccinate against dengue (n = 377).

Variable	Intention to Vaccinate [n (%)]		*p*-Value	OR (95% CI)
	Hesitate and No (n = 142)	Yes (n = 235)		
*Sex*			0.25	1.27 (0.84–1.95)
Male	66 (41.0)	95 (59.0)		
Female	76 (35.2)	140 (64.8)		
*Education level*			<0.01 *	3.23 (1.73–6.05)
Low	30 (62.5)	18 (37.5)		
High	112 (34.0)	217 (66.0)		
*Income*			0.09	1.52 (0.94–2.44)
Low (<IDR 5,000,000)	42 (45.2)	51 (54.8)		
High (≥IDR 5,000,000)	100 (35.2)	184 (64.8)		
*Occupation*			0.05	1.67 (0.99–2.82)
Non-healthcare worker	117 (40.3)	173 (59.7)		
Healthcare worker	25 (28.7)	62 (71.3)		
*Personal history of dengue*			0.22	1.37 (0.83–2.25)
No	112 (39.4)	172 (60.6)		
Yes	30 (32.3)	63 (67.7)		
*Family history of dengue*			<0.01 *	2.24 (1.38–3.62)
No	112 (43.2)	147 (56.8)		
Yes	30 (25.4)	88 (74.6)		
*Knowledge level of dengue*			0.02 *	1.84 (1.11–3.05)
Poor	38 (49.4)	39 (50.6)		
Good	104 (34.7)	196 (65.3)		
*Attitude toward vaccine*			<0.01 *	9.57 (4.97–18.44)
Poor	51 (79.7)	13 (20.3)		
Good	91 (29.1)	222 (70.9)		
*Fear of dengue*			<0.01 *	4.18 (2.67–6.57)
No	102 (53.4)	89 (46.6)		
Yes	40 (21.5)	146 (78.5)		
*Worry of adverse event*			<0.01 *	3.67 (2.11–6.37)
Worry	123 (45.1)	150 (55.0)		
Not worry	19 (18.3)	85 (81.7)		
*Willingness to pay*			<0.01 *	8.98 (5.14–15.69)
Do not want to pay	124 (54.9)	102 (45.1)		
Willing to pay	18 (11.9)	133 (88.1)		

IDR, Indonesian Rupiah; OR, odds ratio; CI, confidence interval. Asterix sign indicated *p*-value less than 0.05. Based on stated answers in a category of “intention to vaccinate against dengue” in Table 1, while respondents with “strongly agree” and “agree” answers were summed into a group of “Yes”, respondents with “not sure” “disagree” and strongly disagree” answers were summed into a group of “Hesitate and No” in this table. *High education level* refers to a respondent who hold a university degree. *Personal history of dengue* refers to respondent’s direct encounter with dengue infection. *Family history of dengue* refers to occurrence of dengue infection among a respondent’s family members. *Knowledge level of dengue* refers to extent and accuracy of a respondent’s understanding on dengue infection. *Attitude toward vaccine* refers to a respondent’s opinion toward receiving the dengue vaccine. *Fear of dengue* refers to a respondent’s anxiety or concern of contracting dengue and/or experiencing its health consequences. *Worry of adverse event* refers to a respondent’s concern or fear about possible side effects that might occur after receiving the dengue vaccine. *Willingness to pay* refers to a respondent’s attitude, regarding the cost and financial responsibility associated with receiving the dengue vaccine.

**Table 3:** Significant risk factors associated with hesitancy to vaccinate based on multivariable logistic regression analysis.

Variable	Intention to Vaccinate [n (%)]		Beta Coefficient	*p*-Value	OR (95% CI)
	Hesitate and No (n = 142)	Yes (n = 235)			
*Willingness to pay*			2.024	<0.01	
Do not want to pay	124 (54.9)	102 (45.1)			7.57
Willing to pay	18 (11.9)	133 (88.1)			(4.06–14.10)
*Attitude toward vaccine*			1.430	<0.01	
Poor	51 (79.7)	13 (20.3)			4.17
Good	91 (29.1)	222 (70.9)			(1.96–8.87)
*Education level*			1.313	<0.01	
Low	30 (62.5)	18 (37.5)			3.72
High	112 (34.0)	217 (66.0)			(1.61–8.59)
*Fear of dengue*			0.933	<0.01	
No	102 (53.4)	89 (46.6)			2.54
Yes	40 (21.5)	146 (78.5)			(1.49–4.34)
*Worry of adverse event*			0.929	<0.01	
Worry	123 (45.1)	150 (55.0)			2.53
Not worry	19 (18.3)	85 (81.7)			(1.34–4.76)

OR, odds ratio; CI, confidence interval. Based on stated answers in a category of ‘intention to vaccinate against dengue’ in Table 1, while respondents with ‘strongly agree’ and ‘agree’ answers were summed into a group of ‘Yes’, respondents with ‘not sure’, ‘disagree’, and ‘strongly disagree’ answers were summed into a group of ‘Hesitate and No’ in this table. *High education level* refers to a respondent who holds a university degree. *Attitude toward vaccine* refers to a respondent’s opinion toward receiving the dengue vaccine. *Fear of dengue* refers to a respondent’s anxiety or concern of contracting dengue and/or experiencing its health consequences. *Worry of adverse event* refers to a respondent’s concern or fear about possible side effects that might occur after receiving the dengue vaccine. *Willingness to pay* refers to a respondent’s attitude, regarding the cost and financial responsibility associated with receiving the dengue vaccine.

## Data Availability

Data are available upon request to the first or corresponding author.

## Supplementary Materials

The following supporting information can be downloaded at: https://www.mdpi.com/article/10.3390/vaccines13121178/s1, Supplementary File: Research questionnaire on "factors related to willingness to receive dengue vaccination among workers"; Figure S1: Distribution of the study participants; Table S1: Characteristics of the study participants through stratification West Java versus Non-West Java.

## Abbreviations

The following abbreviations are used in this manuscript:

CI	Confidence interval
COVID-19	Coronavirus disease 2019
DENV	Dengue virus
OR	Odds ratio
WHO	World Health Organization
