# Supplementary material for: Vaccine Hesitancy Toward Dengue Immunization Among Indonesian Office Workers: A Cross-Sectional Study of Perceptions, Barriers, and Trust Factors
Source: Vaccines (Basel). 2025 Nov 21;13(12):1178. doi: 10.3390/vaccines13121178 (PMC12737692; doi:10.3390/vaccines13121178)
Supplement: Supplementary file 1 [file vaccines-13-01178-s001.zip › vaccines-3919599-supplementary.pdf]

SUPPLEMENTARY FILE

English Version

Research questionnaire "Factors related to willingness to receive dengue vaccination among workers"

A. Personal information

1. Name
2. Date of birth
3. Age
4. Gender:
   a. Male
   b. Female
5. Education:
   a. Junior high school or below
   b. Senior high school
   c. Bachelor's degree
6. Occupation:
   a. Private employee
   b. Civil employee
   c. Healthcare worker
   d. Teacher or lecturer
   e. Entrepreneur
7. Income:
   a. < IDR 5,000,000
   b. IDR 5,000,000 – 10,000,000
   c. IDR 10,000,000 - 25,000,000
   d. > IDR 25,000,000

B. Factors related to willingness to receive dengue vaccine

8. Have you ever had dengue fever?
   a. Yes
   b. No
9. Has anyone in your family had dengue fever?
   a. Yes
   b. No
10. Does your company support dengue vaccination for employees?
    a. Yes
    b. No
11. Please state your current workplace and residential location (city/regency, province)
12. I want to get the dengue vaccine:
    a. Strongly disagree
    b. Disagree
    c. Neutral/not sure
    d. Agree
    e. Strongly agree
13. I feel it is reasonable to pay if I want to receive the dengue vaccine:
    a. Strongly disagree
    b. Disagree
    c. Neutral/not sure
    d. Agree
    e. Strongly agree
14. What is a reasonable price to pay for a dengue vaccine?
    a. <IDR 100,000
    b. IDR 100,000 – 250,000

c. IDR 250,000 – 500,000
d. IDR 500,000 – 1,000,000

C. Knowledge (check your answers in the appropriate column)

No.	Question	True	False	Not Sure
15	Is dengue fever caused by mosquitoes?			
16	Do all mosquitoes around us carry the dengue virus?			
17	Is it only female mosquitoes that suck blood?			
18	Do dengue-spreading mosquitoes lay eggs in stagnant water?			
19	Are dengue-spreading mosquitoes active during the day and late afternoon?			
20	Can dengue fever only be spread through mosquito bites?			
21	Must dengue fever symptoms include red spots on the skin?			
22	Can dengue fever be cured by taking paracetamol?			
23	Can dengue fever be prevented by drinking guava juice?			
24	Does wearing light-coloured clothing and covering the whole body prevent mosquito bites?			
25	Is dengue fever prevention done by "Covering, Disposing, and Draining"?			
26	Can dengue fever be prevented with vaccination?			

D. Attitudes toward dengue prevention (check your answers in the appropriate column)

No.	Question	Strongly Disagree	Disagree	Not Sure	Agree	Strongly Agree
27	Dengue fever is very dangerous and fatal					
28	I am at risk of contracting dengue fever					
29	Following all prevention protocols can prevent me from contracting dengue fever					
30	Bekasi regency is a high-risk area for dengue fever					
31	I trust healthcare facilities					
32	I am worried about contracting dengue fever					
33	I am not worried about the side effects of the dengue vaccine					
34	Vaccination is important for preventing disease					
35	Religion allows me to get vaccinated as a health effort					
36	Dengue vaccination can prevent dengue fever					
37	Dengue fever vaccine is safe					

##########

<u>Versi Bahasa Indonesia</u>

Kuesioner penelitian "Faktor yang berhubungan dengan keinginan mendapatkan vaksin dengue bagi pekerja"

A. Data pribadi

1. Nama
2. Tanggal lahir
3. Usia
4. Jenis kelamin:
   a. Laki-laki
   b. Perempuan
5. Pendidikan:
   a. Lulus SMP atau kurang
   b. Lulus SMA
   c. Lulus Perguruan tinggi
6. Pekerjaan:
   a. Karyawan swasta
   b. Pegawai negeri sipil
   c. Tenaga kesehatan
   d. Pengajar
   e. Wiraswasta
7. Penghasilan:
   a. < Rp 5.000.000
   b. Rp 5.000.000 – 10.000.000
   c. Rp 10.000.000 – 25.000.000
   d. > Rp 25.000.000

B. Hal-hal yang berhubungan dengan keinginan mendapatkan vaksin dengue atau vaksin demam berdarah dengue/DBD

8. Apakah bapak/ibu pernah kena DBD?
   a. Ya
   b. Tidak
9. Apakah ada anggota keluarga yang terkena DBD?
   a. Ya
   b. Tidak
10. Apakah perusahaan mendukung vaksinasi pada karyawan?
   a. Ya
   b. Tidak
11. Sebutkan lokasi tempat kerja dan tempat tinggal Anda saat ini (kota/kabupaten, provinsi)
12. Saya ingin mendapat vaksin DBD:
   a. Sangat tidak setuju
   b. Tidak setuju
   c. Netral/tidak pasti
   d. Setuju
   e. Sangat setuju
13. Saya merasa wajar untuk membayar jika ingin mendapat vaksin DBD:
   a. Sangat tidak setuju
   b. Tidak setuju
   c. Netral/tidak pasti
   d. Setuju
   e. Sangat setuju
14. Berapakah harga yang pantas jika saya perlu membayar vaksin DBD?
   a. <Rp 100.000
   b. Rp 100.000 – 250.000
   c. Rp 250.000 – 500.000

d. Rp 500.000 – 1.000.000

C. Pengetahuan (berikan jawaban Anda di kolom yang sesuai)

No	Pertanyaan	Benar	Salah	Tidak pasti
15	Apakah penyakit demam berdarah disebabkan oleh nyamuk?			
16	Apakah semua nyamuk yang ada di sekitar kita membawa virus DBD?			
17	Apakah hanya nyamuk betina yang menghisap darah?			
18	Apakah nyamuk yang menyebar DBD bertelur di air yang menggenang?			
19	Apakah nyamuk yang menyebar DBD aktif menggigit pada siang dan sore hari?			
20	Apakah DBD hanya dapat menyebar di masyarakat akibat gigitan nyamuk?			
21	Apakah gejala demam berdarah harus ada bintik merah di kulit?			
22	Apakah DBD dapat disembuhkan dengan minum paracetamol?			
23	Apakah DBD dapat dicegah dengan minum jus jambu biji?			
24	Apakah mengenakan pakaian dengan warna cerah dan menutupi seluruh tubuh mencegah gigitan nyamuk?			
25	Apakah pencegahan DBD adalah dengan Gerakan 3M, yaitu "Menutup, Membuang dan Menguras"?			
26	Apakah DBD dapat dicegah dengan vaksinasi?			

D. Sikap terhadap pencegahan DBD (berikan jawaban Anda di kolom yang sesuai)

No.	Pertanyaan	Sangat tidak setuju	Tidak setuju	Tidak pasti	Setuju	Sangat setuju
27	DBD sangat berbahaya dan bersifat fatal					
28	Saya berisiko tertular DBD					
29	Mengikuti semua protokol pencegahan dapat mencegah diri saya terkena demam berdarah dengue					
30	Kabupaten Bekasi merupakan area risiko tinggi DBD					
31	Saya percaya pada fasilitas kesehatan					
32	Saya cemas akan kena DBD					
33	Saya tidak khawatir dengan efek samping vaksin DBD					
34	Vaksinasi penting untuk mencegah penyakit					
35	Agama memperbolehkan saya untuk melakukan vaksinasi sebagai upaya menjaga kesehatan					
36	Vaksinasi dengue dapat mencegah DBD					
37	Vaksin DBD aman					

FIGURE S1

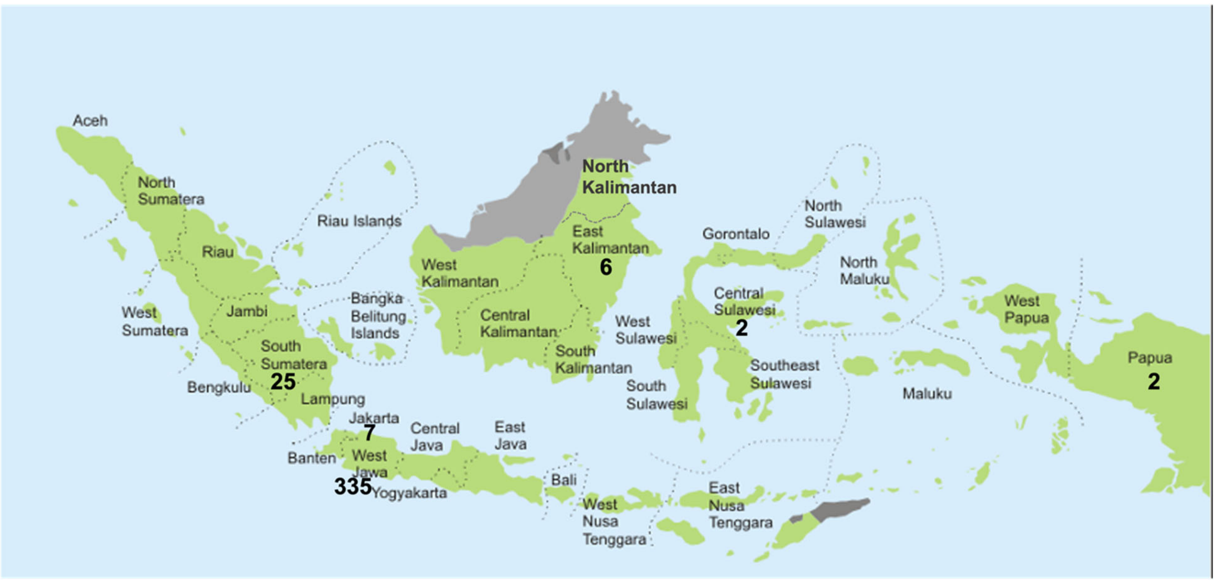

Figure S1. Distribution of the study participants. Total there were 377 participants, located in South Sumatra (n=25), Jakarta (n=7), West Java (n=335), East Kalimantan (n=6), Central Sulawesi (n=2) and Papua (n=2).

TABLE S1

Characteristics of the study participants through stratification West Java versus Non-West Java.

Variables	Total (n=377)	West Java (n=335)	Non-West Java (n=42)	p-value
Age in year [Mean ± SD (Min – Max)]	38.2 ± 9.67 (18 – 60)	38.03 ± 9.76 (18 – 60)	39.5 ± 8.97 (24 – 60)	0.355
Sex [n (%)]				
Female	216 (57.3)	190 (56.7)	26 (61.9)	0.522
Male	161 (42.7)	145 (43.3)	16 (38.1)	
Education [n (%)]				
Elementary-high school	48 (12.7)	45 (13.4)	3 (7.1)	0.249
University	329 (87.3)	290 (86.6)	39 (92.9)	
Income [n (%)]				
< IDR 5,000,000	93 (24.7)	81 (24.2)	12 (28.6)	0.395
IDR 5,000,000 – 10,000,000	193 (51.2)	174 (51.9)	19 (45.2)	
IDR 10,000,000 – 25,000,000	66 (17.5)	56 (16.7)	10 (23.8)	
> IDR 25,000,000	25 (6.6)	24 (7.2)	1 (2.4)	
Occupation [n (%)]				
Non-healthcare worker	290 (76.9)	255 (76.1)	35 (83.3)	0.577
Healthcare worker	87 (23.1)	80 (23.9)	7 (16.7)	
Personal history of dengue [n (%)]				
No or do not know	284 (75.3)	251 (74.9)	33 (78.6)	0.605
Yes	93 (24.7)	84 (25.1)	9 (21.4)	
Family history of dengue [n (%)]				
No	259 (68.7)	230 (68.7)	29 (69.1)	0.959
Yes	118 (31.3)	105 (31.3)	13 (30.9)	
Knowledge level of dengue [n (%)]				
Poor	77 (20.4)	68 (20.3)	9 (21.4)	0.864
Good	300 (79.6)	267 (79.7)	33 (78.6)	
Attitude toward dengue vaccine [n (%)]				
Poor	64 (17.0)	50 (14.93)	14 (33.33)	0.003
Good	313 (83)	285 (85.07)	28 (66.67)	
Intention to vaccinate against dengue [n (%)]				
Strongly agree	50 (13.3)	45 (13.4)	5 (11.9)	0.241
Agree	185 (49.1)	169 (50.5)	16 (38.1)	
Not sure	126 (33.4)	108 (32.2)	18 (42.8)	
Disagree	10 (2.7)	9 (2.7)	1 (2.4)	
Strongly disagree	6 (1.6)	4 (1.2)	2 (4.8)	
Opinion about paying the vaccine [n (%)]				
Strongly agree	19 (4.2)	17 (5.1)	2 (4.8)	0.896
Agree	132 (35.0)	119 (35.5)	13 (30.9)	
Not sure	163 (43.2)	142 (42.4)	21 (50.0)	
Disagree	47 (12.5)	43 (12.8)	4 (9.5)	
Strongly disagree	16 (4.2)	14 (4.2)	2 (4.8)	

IDR, Indonesian Rupiah; SD, standard deviation. Results between "West Java" and "Non-West Java" were statistically compared per category. While the Mann–Whitney U test was used for a numerical variable (i.e., age), Chi-square tests were used for categorical variables. Statistical significance was defined as p-value <0.05.
